# Supplementary material for: Light-Weight Wearable Gyroscopic Actuators Can Modulate Balance Performance and Gait Characteristics: A Proof-of-Concept Study
Source: Healthcare (Basel). 2023 Oct 27;11(21):2841. doi: 10.3390/healthcare11212841 (PMC10647239; doi:10.3390/healthcare11212841)
Supplement: Supplementary file 1 [file healthcare-11-02841-s001.zip › healthcare-2560963-supplementary.pdf]

GyroPack - Device description

Bram Sterke Cor Meijneke Giel Hermans
Katherine Poggensee Wouter Gregoor Cornel Weststeijn
Evelien van Pruijssen Daniel Lemus Perez Heike Vallery

February 2023

The GyroPack is a backpack that is built to assist, or intentionally perturb, balance during standing and walking. It is configured and worn similar to a regular backpack. The backpack can exert forces/moments onto a person's trunk. These moments are generated by two control moment gyroscopes (CMG)[3].

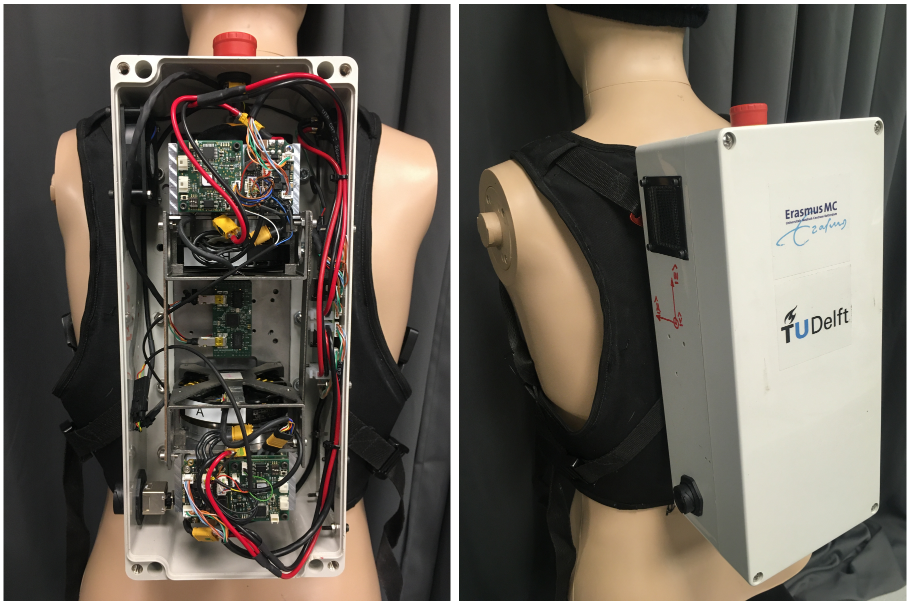

Figure S1: Left: uncovered GyroPack backpack, showing two control moment gyroscopes with gimbal axes aligned with the vertical axis of the trunk. The inertial measurement unit is located in the center of the box. Right: GyroPack with protective cover.

1 Working principle

The main principle of operation of the device is based on control moment gyroscopes (CMG). CMGs contain a fast-spinning flywheel and a gimbal motor that can change the orientation of this flywheel. In recent years we have miniaturized the actuators such that each actuator is an independent unit and only weighs about 1.5 kg[3]. The GyroPack contains two miniaturized actuators. The change of orientation imposed by the gimbal motor, combined with the angular momentum of the flywheels, causes a free moment, or torque, that is exerted onto the system the CMG is attached to, which in this study is the human upper body. We use algorithms that can reliably deal with actuator limitations without causing undesired disturbances on the user[4, 1]. The gimbals are controlled in such a way that the torque exerted on the trunk is proportional and opposite to the trunk's angular velocity, which effectively lets the system damp rotational motion of the wearer. This damping has been shown to make balancing easier for unimpaired subjects and individuals post-stroke[2].

2 Main components

Each CMG consists of a flywheel and a gimbal which together generate the change in angular momentum that is exerted onto the person. The GyroPack also contains an inertial measurement unit (IMU) to determine the orientation and accelerations of the CMG. These components are controlled and read through a set of drives.

The system is powered through a separate power box. This box takes care of the filtering and electrical separation of electrical signals, which is important for the electrical safety of the device. This box also contains the emergency stop (E-stop) button, which, when pressed, cuts all the power to the device. This acts as a final safety measure, allowing the operator to physically stop all of the backpack's activity.

The control algorithm of the backpack runs on a desktop computer located near the operator. This contains the logic input/output modules and the EtherCAT master, i.e. the controller that sends commands to the drives in the CMG. The base station is also the computer where the operator can input settings and commands through the user interface (a computer screen) and where all the device data are logged and stored.

The operating system of the GyroPack consists of a real-time compatible target PC which runs a Simulink model using the TwinCAT 3 platform. For the core of the damping algorithm we used the directional singularity escape and avoidance (DSEA) controller as described in Section 5.

3 Technical Specifications

Table S1 describes the technical specifications of the GyroPack as a whole, con-taining two CMGs with both gimbals aligned with the vertical axis of the trunk.

Table S1: Device specifications of GyroPack

Dimensions (H x W x D)	40 x 30 x 15 cm (Cover containing two CMGs)
	50 x 70 x 17 cm (Entire backpack/harness)
Weight	4.9 kg
Power supply	230V 1P AC Mains
Max. torque (peak)	30 Nm (for 0.1 seconds)
Amount of CMGs	2
Planes of assistance	Frontal and sagittal
Control Modes	Damper, Placebo

Table S2 describes the technical specifications of each single CMG located within the GyroPack.

Table S2: Device specifications o f m ini c ontrol m oment g yroscope u sed i n Gy-roPack

Gimbal torque max	2.14	Nm
Gimbal velocity max	300	RPM
Gimbal accel max	20	Rev/s^2
FW vel max	20.000	RPM
FW vel rate limiter	Unlimited	
Iw (of flywheel)	0.00022651	kgm^2
IMU temp offset	0	
IMU temp sensitivity	333.87	LSB/degC
G encoder resolution	2ˆ14	

4 High-level control

The main components of the high-level control loop are; the inertial measuremnet unit (IMU), directional singularity escape and avoidance (DSEA), two control moment gyroscopes (CMGs), user inputs, and constant parameters. Where $\boldsymbol{\omega}$ is the trunk angular rate, $\mathbf{R}_{B/G}$ is the rotation matrix that maps the IMU outputs to the coordinate frame of the trunk, k_{d} is the damping gain, J_{s} denotes the flywheel inertia, mode sets the control mode (i.e. damping or placebo), k_{plac} is the gain relating trunk angular rate to gimbal motion during the placebo mode, Ω_i are the flywheel speeds, γ_i denotes the current gimbal positions, $\boldsymbol{\tau}_{\mathrm{ref}}$ is the reference torque, and $\dot{\boldsymbol{\gamma}}_{\mathrm{ref}}$ is the gimbal reference velocity vector.

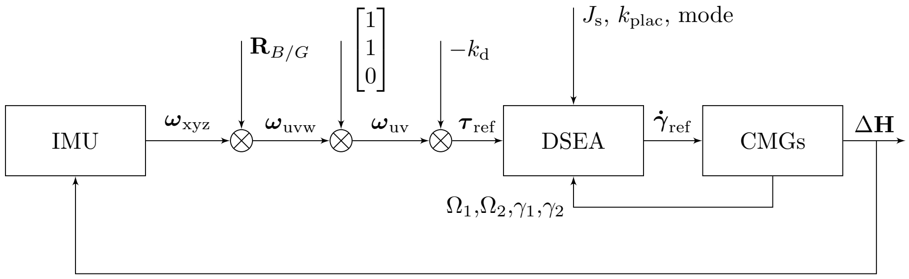

Figure S2: Flowchart displaying high-level controller of GyroPack. Where $\boldsymbol{\omega}$ is the trunk angular rate, $\mathbf{R}_{B/G}$ is the rotation matrix of the IMU to body coordinate frames, k_{d} is the damping gain, J_{s} denotes the flywheel inertia, k_{plac} is the gain relating trunk angular rate to gimbal motion during the placebo mode, Ω is the flywheel s peed, m ode s ets t he c ontrol m ode (i.e. d amping or placebo), γ denotes the current gimbal positions, $\boldsymbol{\tau}_{\mathrm{ref}}$ is the reference torque, and $\dot{\boldsymbol{\gamma}}_{\mathrm{ref}}$ is the gimbal reference velocity vector.

5 DSEA

The DSEA function at the core of the high-level controller is based on Valk et al.(2018), adapted to contain two CMGs with singularity robust control, as described by Berry et al.(2016) [1, 4].

The matrix $\mathbf{F}$ contains the contains the direction vectors of the gyroscopic moments (neglecting moments induced by human movement), based on gimbal positions γ_1 and γ_2.

$$\mathbf{F} = \begin{bmatrix} -\sin(\gamma_1) & -\sin(\gamma_2) \\ \cos(\gamma_1) & \cos(\gamma_2) \end{bmatrix} \tag{1}$$

A damping term (α) is calculated based on the smallest singular value (σ_m) of the gimbal frame vectors ($\mathbf{F}$) to determine closeness to a singularity. It uses

damping parameters σ_{min} and η to determine the damping behaviour.

$$\alpha = \sigma_{\text{min}}^2 e^{\eta(\sigma_{\text{min}}^2 - \sigma_m^2)} \tag{2}$$

The damped pseudo-inverse solution to the optimization $\mathbf{F}^\circ$ is calculated, based on the damping term α and the (last) left singular vector ($\boldsymbol{u}_{\text{m}}$), spanning the m-dimensional moment output space of the singular value decomposition of the F matrix.

$$\mathbf{F}^\circ = \mathbf{F}^\mathsf{T}(\mathbf{F}\mathbf{F}^\mathsf{T} + \alpha \boldsymbol{u}_{\text{m}} \boldsymbol{u}_{\text{m}}^\mathsf{T})^{-1} \tag{3}$$

The desired gimbal velocities $\dot{\boldsymbol{\gamma}}_s$ are calculated based on the desired torque and two tuning parameters d_0 and ζ, as described in equation 46 of Valk et al. (2018). The per-gimbal dimensionless escape potential ($\boldsymbol{d}_i$) describes the gimbal rate magnitude, which pushes the gimbal from its anti-saturated state. However, we have simplified the choice of escape direction, similar to Berry et al. (2016), always creating opposed escape velocities.

$$d_i = \begin{cases} \boldsymbol{h}_i^\mathsf{T} \boldsymbol{\tau}_{\text{ref}} & \text{if } \boldsymbol{h}_i^\mathsf{T} \boldsymbol{\tau}_{\text{ref}} < 0 \\ 0 & \text{otherwise} \end{cases} \tag{4}$$

$$\dot{\boldsymbol{\gamma}}_s = \begin{bmatrix} 1 \\ -1 \end{bmatrix} d_i \frac{d_0 \| \frac{\boldsymbol{\tau}_{\text{ref}}}{\boldsymbol{\tau}_{\text{max}}} \|^\zeta}{(\|\boldsymbol{\tau}_{\text{ref}}\|^3 + \boldsymbol{\tau}_{\text{max}}^3)} \cdot \sqrt{\sum_{j=1}^{2} (1 - \boldsymbol{z}_j)^2 (\boldsymbol{u}_j^\mathsf{T} \boldsymbol{\tau}_{\text{ref}})^2} \tag{5}$$

Where $\boldsymbol{h}_1$ and $\boldsymbol{h}_2$ denotes the direction of angular momentum of gimbals, $\boldsymbol{\tau}_{\text{ref}}$ is the desired torque, $\boldsymbol{\tau}_{\text{ref}}$ is the maximally allowed torque, and $\boldsymbol{z}_j$ is the result of the sigmoidal function:

$$z_j = \frac{\sigma_j^2}{\sigma_j^2 + \sigma_{\text{acp}}^2 e^{\eta(\sigma_{\text{acp}}^2 - \sigma_j^2)}} \tag{6}$$

Where σ_{acp} is a predefined acceptable value that facilitates tracking with acceptable gimbal rates, σ_j is the jth singular value, and η is a constant defining the stepness of the sigmoidlike function.

Lastly, the reference gimbal velocities $\dot{\boldsymbol{\gamma}}_{\text{ref}}$, which are sent to the gimbal drives, are calculated.

$$\dot{\boldsymbol{\gamma}}_{\text{ref}} = \frac{\mathbf{F}^\circ \boldsymbol{\tau}_{\text{ref}}}{J_{\text{s}} \Omega} + (\mathbf{I}_2 - \mathbf{F}^\circ \mathbf{F}) \dot{\boldsymbol{\gamma}}_{\text{s}} \tag{7}$$

where $\mathbf{I}_2$ is a 2x2 identity matrix, J_{s} denotes the flywheel inertia, $\boldsymbol{\Omega}$ contains the flywheel velocity vectors of both flywheels, and $\mathbf{F}^\circ$ is the right pseudo-inverse with numerical filtering.

6 Placebo

During the placebo controller the gimbals were maintained at an angle of 180°as follows:

$$\gamma_{\mathrm{d}} = \sin(\gamma_2 - \gamma_1) \tag{8}$$

$$\dot{\gamma}_{\mathrm{p}} = k_{\mathrm{plac}} \sqrt{\omega_{\mathrm{u}}^2 + \omega_{\mathrm{v}}^2} \tag{9}$$

$$\dot{\boldsymbol{\gamma}}_{\mathrm{ref}} = \dot{\gamma}_{\mathrm{p}} \begin{bmatrix} -1 \\ 1 \end{bmatrix} \frac{\gamma_{\mathrm{d}}}{2} \tag{10}$$

Where γ_2 and γ_1 are the gimbal positions, ω_{u} and ω_{v} is the vector filled with the $\hat{u}$ and $\hat{v}$ components of the trunk angular rate of the human, and k_{plac} is a constant gain.

References

[1] Andrew Berry, Daniel Lemus, Robert Babuška, and Heike Vallery. Directional Singularity-Robust Torque Control for Gyroscopic Actuators. *IEEE/ASME Transactions on Mechatronics*, 21(6):2755–2763, December 2016. Citation Key Alias: berry_directional_2016a.

[2] Daniel Lemus, Andrew Berry, Saher Jabeen, Chandrasekaran Jayaraman, Kristen Hohl, Frans C. T. van der Helm, Arun Jayaraman, and Heike Vallery. Controller synthesis and clinical exploration of wearable gyroscopic actuators to support human balance. *Scientific Reports*, 10(1):10412, December 2020.

[3] Cory Meijneke, Bram Sterke, Giel Hermans, Wouter Gregoor, Heike Vallery, and Daniel Lemus. Design and Evaluation of Pint-Sized Gyroscopic Actuators. In *2021 IEEE/ASME International Conference on Advanced Intelligent Mechatronics (AIM)*, pages 454–461, Delft, Netherlands, July 2021. IEEE.

[4] Laurens Valk, Andrew Berry, and Heike Vallery. Directional Singularity Escape and Avoidance for Single-Gimbal Control Moment Gyroscopes. *Journal of Guidance, Control, and Dynamics*, 41(5):1095–1107, May 2018.
